# Plant RNA binding proteins for control of RNA virus infection

**DOI:** 10.3389/fphys.2013.00397

**Published:** 2013-12-31

**Authors:** Sung Un Huh, Kyung-Hee Paek

**Affiliations:** College of Life Sciences and Biotechnology, Korea UniversitySeoul, South Korea

**Keywords:** antiviral activity, plant defense response, plant RNA virus, RNA binding protein, virus infection

## Abstract

Plant RNA viruses have effective strategies to infect host plants through either direct or indirect interactions with various host proteins, thus suppressing the host immune system. When plant RNA viruses enter host cells exposed RNAs of viruses are recognized by the host immune system through processes such as siRNA-dependent silencing. Interestingly, some host RNA binding proteins have been involved in the inhibition of RNA virus replication, movement, and translation through RNA-specific binding. Host plants intensively use RNA binding proteins for defense against viral infections in nature. In this mini review, we will summarize the function of some host RNA binding proteins which act in a sequence-specific binding manner to the infecting virus RNA. It is important to understand how plants effectively suppress RNA virus infections via RNA binding proteins, and this defense system can be potentially developed as a synthetic virus defense strategy for use in crop engineering.

## Introduction

RNA binding proteins (RBPs) play critical roles in post-transcriptional gene regulation by controlling splicing, polyadenylation, mRNA stability, RNA trafficking and translation (Moore, 2005; Glisovic et al., 2008; Pallas and Gomez, 2013). Furthermore, some RBPs work as RNA chaperones (Kang et al., 2013). RBPs have a specialized RNA binding domain (RBD) which can bind to target RNAs. Examples of RBDs include RNA recognition motif (RRM), Pumilio/FBF (PUF) domain, K Homology (KH) domain, and double-stranded RNA binding domain (DS-RBD) (Maris et al., 2005; Lunde et al., 2007; Quenault et al., 2011). Some RBPs have been known involved in plant innate immunity, although limited RBPs have been characterized in detail in plant defense (Fu et al., 2007; Qi et al., 2010; Lee et al., 2012a,b).

Infection of host cells by plant RNA viruses has long been puzzling because they usually encode only a few proteins. However, plant RNA viruses suppress host innate immunity or utilize host proteins for their successful replication and movement (Laliberte and Sanfacon, 2010; Pallas and Garcia, 2011). On the other hand, plants have evolved a variety of strategies to ward off virus infection. When a plant virus is infected into host cells, mainly by mechanical wounding or damage caused by insects, RNAs of the plant RNA virus are exposed from coat proteins and first induce cellular membrane-associated structures for replication, mainly because naked RNA is susceptible to degradation by RNases. Plants have evolved innate immunity toward RNAs of infecting RNA viruses. RNA interference (RNAi), also called post-transcriptional gene silencing (PTGS), is one aspect of the viral RNA-targeting host innate immunity. RNAi has an important role in protecting cells against plant RNA virus infection via sequence-specific binding of small interfering (si) RNAs (Simon-Mateo and Garcia, 2011). RNA of infecting plant RNA viruses is a critical target of suppression in terms of host defense.

Plant RBPs are involved in the viral RNA-targeted host innate immunity against plant RNA virus infection (Zhu et al., 2007; Fujisaki and Ishikawa, 2008; Huh et al., 2013). Sequence-specific recognition of a plant RNA binding protein is likely to be important for the regulation of specific targets, and has been linked to many developmental processes, as well as biotic and abiotic stresses (Lorkovic, 2009; Woloshen et al., 2011; Ambrosone et al., 2012). Thus, host RNA binding proteins might be directly or indirectly involved in viral RNA-targeted defense against infecting RNA virus at the transcriptional/translational level. However, the functions of some of these RBPs against virus infection have yet to be determined. Here, we provide a brief overview focused on host RBP-virus RNA direct/indirect interactions.

## Host RBPs contribute to host immunity via host RNA regulation or viral RNA degradation against plant RNA virus infection

**Figure 1:**
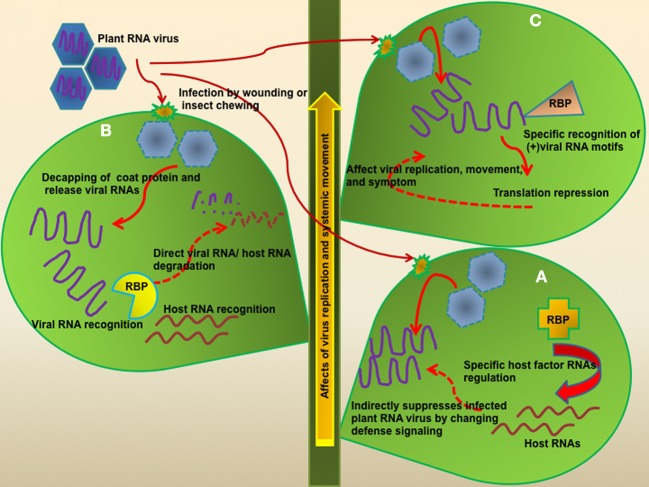
**Modes of defense function of RBPs against plant RNA virus infection**. Most plant RNA viruses enter the host cell by wounding or insect damage. In the host cell, viral RNAs are released from a coat protein and then localize to the membrane-like structures in order to protect naked viral RNA. How plants recognize plant virus infection is still unknown. Plants positively set up viral RNA-targeted defense system using host RBPs. **(A)** Some RBPs regulate plant defense signaling pathway genes at posttranscriptional levels and these can control a variety of pathogens in addition to viruses. The direct target RNAs of most RBPs were still not identified. **(B)** PR10 family proteins have RNase activity, as well as anti-viral, anti-bacterial, and anti-fungal activity. Furthermore, PR10 proteins are involved in development and abiotic stress. One of them, CaPR10, can recognize and cleave TMV viral RNA. However, this recognition mechanism of PR10 for viral RNA/host RNA is not clear, but they might need the helper protein(s) for determination of their specific target or other function. **(C)** Some RBPs have a specific binding motif of target viral RNA but other RBPs may bind to some specific nucleotide rich regions or recognize specific RNA structures. Nevertheless, these RBPs offer translational repression of viral RNA and affect viral replication, movement, and symptoms.

Several RBPs containing RNA binding domains have been implicated in plant innate immunity (Woloshen et al., 2011; Staiger et al., 2013). These RBPs could function in sequence-specific or non-specific manners. Many cases of RBP-mediated plant defense strategy exhibited that RBPs targeted host RNA at mRNA levels, and controlled defense signaling pathways. For example, *Arabidopsis thaliana* RNA-binding protein-defense related 1 (AtRBP-DR1) positively contributes to hemibiotrophic pathogen defense, and possibly regulates genes involved in the salicylic acid (SA) signaling pathway in the cytosolic region, via direct binding of target RNAs (Qi et al., 2010). Interestingly, overexpression of glycine-rich RNA-binding protein 7 (AtGRP7), which contains an RNA-recognition motif (RRM), conferred plant defense against pathogens including *Pseudomonas syringae* pv. *tomato* DC3000 (*Pst* DC3000), necrotrophic bacterium *Pectobacterium carotovorum* SCC1 as well as the biotrophic virus *Tobacco mosaic virus* (Lee et al., 2012b). The comparison of global transcript profiling between the wild type and AtGRP7-overexpressing transgenic plants revealed that approximately 300 transcripts, including those involved in circadian clock, stress response, ribosome function, and RNA metabolism, were regulated by AtGRP7 (Streitner et al., 2010). However, direct target RNAs of these RBPs and RNA binding motifs were not identified. The molecular mechanisms of RBPs-mediated defense response against diverse pathogens are largely unknown, but it is possible that RBPs generally regulate defense signaling-related genes at posttranscriptional/translational levels upon pathogen infections (Figure 1A).

Whilst RBPs can broadly regulate host innate immunity against a variety of pathogen infections, some RBPs directly participate in virus resistance via binding to viral RNAs. For instance, the pathogenesis-related protein PR10 (PR10) family has been identified as ribonuclease-like PR proteins (Fernandes et al., 2013). These proteins have highly conserved regions including a specific domain (KAXEXYL), and the P-loop motif (GXGGXGXXK), which is known as an RNA binding site, but it is not clear whether these sites offer specific binding affinity to target RNA. One of the PR10 family, CaPR10, which was isolated from hot pepper (*Capsicum annuum*), was determined to be directly involved in plant defense against viral RNA infection, and showed enhanced ribonucleolytic activity to viral RNAs upon infection (Park et al., 2004). Another PR10 family member, TcPR10 from *Theobroma cacao*, showed antifungal activity against *Moniliophthora perniciosa*, and had *in vivo* ribonuclease activity (Pungartnik et al., 2009). Even though PR10 proteins function as RBPs it is still not clear if they are specifically involved in RNA virus defense, as PR10 is also known to be involved in defense functions during a variety of abiotic and biotic stresses (Srivastava et al., 2004; Chen et al., 2010). Although the effects of the PR10 family seem to be non-specific, it is possible that the PR10 family could have helper proteins for specific binding of target RNAs, such as viral or host RNAs (Figure 1B). Recently, CaPR10 via interacting with leucine-rich repeat 1 (LRR1) protein, exhibited an enhanced HR-like cell death phenotype, and defense signaling was activated. However, the CaPR10-LRR1 interaction-mediated defense mechanism is still not clear (Choi et al., 2012). How the PR10-LRR1 interaction activates plant defense and how CaPR10 recognizes the host RNAs remain to be solved. Thus, it will be interesting to identify the host RNA targets and more binding partner proteins of PR10 during plant defense responses.

## Host RBPs contribute to host immunity via translational repression of plant RNA virus

Upon plant virus infection, plants recognize the invading RNA virus by unknown detection systems, and the plant innate immune system is activated to suppress viral infection. As one component of the viral RNA-targeted defense system, RBPs directly bind to the RNA of the infecting plant RNA virus and affect replication and movement. These direct defense systems control the invading RNA virus effectively. Some RBPs are indeed reported to bind to the plant RNA viruses directly. *Arabidopsis* Pumilio RNA binding protein 5 (APUM5) directly bound to the “UGUA”–containing nucleotides in the 3′ untranslated region (UTR) and internal regions of *Cucumber mosaic virus* (CMV) and suppressed replication of CMV. Furthermore, APUM5 had a function of suppressing translation (Huh et al., 2013). In the mammalian system, Pumilio-fem-3 mRNA binding factor (PUF) RNA-binding protein has been known as a post-transcriptional/translational repressor, via binding to the 3′ UTR regions of its target mRNAs (Wharton and Aggarwal, 2006). PUFs play critical roles in the developmental steps of various eukaryotic organisms (Wickens et al., 2002; Quenault et al., 2011). PUFs contain a defined and highly conserved Pumilio homology domain (PHD) at the C-terminal region. PHD is highly conserved across species, and may be represented as canonical or non-canonical PHD (Quenault et al., 2011). Eight alpha helical repeats of PHD could confer recognition and binding affinity of target RNA (Miller and Olivas, 2011). In yeast, Puf 3, 4, and 5 have over 200 putative target genes which have the conserved “UGUX3-5UA” motif (Gerber et al., 2004). As mentioned above, APUM5 also has the conserved PHD which has RNA binding capacity to some internal regions, as well as 3′ UTR motif (5′-UGUACUUCUA-3′) of CMV RNA 1, RNA 2, and RNA 3, *in vitro* and *in vivo*. Furthermore, APUM5-PHD also bound to the *Nanos response elements* 2 (NRE2) core sequence (5′-UGUACAUA-3′) within the 3′ UTR of *hunchback* mRNA (Huh et al., 2013). *Turnip mosaic virus* (TuMV) also contains putative PHD-binding core motifs in its genome. When TuMV was inoculated in *APUM5*-overexpressing transgenic plants, the transgenic plants exhibited reduced TuMV RNA levels and slightly increased resistance compared to wild-type plant at the early stage (Huh et al., 2013). APUM5 could act as a viral RNA-targeted plant defense protein, and might also regulate unknown host target RNAs in an RNA sequence-specific manner. In mammalian systems, PUFs associate with Ccr4-Pop2p-NOT mRNA deadenylase complex, and then attack the 3′ UTR of target mRNAs (Goldstrohm et al., 2006). However, *Arabidopsis* Pop2p homologs did not interact with APUM5 (Huh and Paek, 2013). Furthermore, the 3′ UTR of CMV forms a tRNA-like structure (TLS) but does not have a poly(A) tail.

Originally, mammalian poly (rC)-binding proteins (PCBPs) were known to contain three hnRNP KH RNA binding domains, and these domains are essential for the multiplication of polioviruses. PCBPs interact with the cytidine-rich RNA region of poliovirus RNA 5′ UTR (Toyoda et al., 2007). Interestingly, a PCBP homolog in *Arabidopsis*, Binding to *Tomato mosaic virus* (ToMV) RNA 1 (BTR1), negatively affects ToMV multiplication, and suppresses the spread of the virus via interaction with the 5′ terminal region, which contains the initiation codon for the ToMV replication proteins (Fujisaki and Ishikawa, 2008). However, this region is not enriched with cytidine residues. Furthermore, it does not have any unique secondary structures or any specific RNA binding motifs for host RBPs (Fujisaki and Ishikawa, 2008). In the reporter assay, BTR1 was determined to work at the translational level but not at the mRNA stability level (Fujisaki and Ishikawa, 2008). BTR1 might act as a translational repressor because BTR1 could affect production of the 130 K and 180 K replication proteins, via direct binding to the viral RNA. The mechanism of the antiviral activity of BTR1 remains to be discovered. It is currently unclear whether BTR1 has specific binding motifs for plant viral RNA or whether BTR1 indirectly affects host innate immunity.

Some RBPs are differentially regulated at the translational or post-transcriptional level depending on the cell type, developmental stage, or biotic/abiotic interactions (Woloshen et al., 2011; Kang et al., 2013). In the antiviral RBPs, *Arabidopsis* dsRNA-binding protein 4 (DRB4), as the dicer-like 4 (DCL4) interacting partner, was involved in antiviral defense against *Turnip yellow mosaic virus* (TYMV) infection (Jakubiec et al., 2012). Upon the virus infection, DRB4 is recruited from the nucleus to the cytoplasm to regulate viral infection (Jakubiec et al., 2012). DRB4 is specifically required for DCL4 activity in cleaving long dsRNA into 21-nt small RNA (Fukudome et al., 2011). Furthermore, DRB4 directly binds to the tRNA-like structure (TLS) which has critical roles for the replication and translation of viral RNA, although TLS does not have any specialized binding motif for dsRNA binding protein but the structure of TLS might confer a possible binding motif. DRB4 may act as a translational repressor of plant RNA viruses as DRB4 suppresses viral RNA translation, but not degradation. However, it remains to be determined whether DBR4 works at RNA or protein level to regulate target host RNA and viral RNA (Figure 1C).

## Conclusions and perspectives

Plant RNA viruses belong to the large group of all known viruses and are responsible for a vast variety of plant diseases. Plant genomes encode hundreds of RBPs, which are believed to bind to specific target mRNAs and affect plant physiology. However, only a few RBPs have been characterized so far. We briefly reviewed some RNA binding proteins which positively regulate plant innate immunity via direct binding to the viral RNAs (Table 1). Normally, plant RNA viruses utilize host RNA binding proteins to form the viral RNA replication complex or to achieve viral RNA protection from the host innate immune system. Nevertheless, other host RBPs could control RNA virus infection at the posttranscriptional level via direct binding to viral RNA. The function of these RBPs is still not clear, in terms how host RBPs-viral RNAs interactions are specifically regulated, or how RBPs affect the fate of viral RNAs. Future studies may provide clearer insights in these areas, especially at the mechanistic level. Furthermore, these host RBPs have the potential to be exploited as new viral RNA-targeted plant defense systems. For instance, PHD of Pumilio RNA binding protein could possibly be engineered for specific binding to the target RNA motif, thereby controlling viral infection. This might lead to a redesigning of plant immunities at posttranscriptional/translational levels (Qiu et al., 2012). A better understanding of RBPs-viral RNA interaction mechanism will contribute to the development of these antiviral systems.

**Table 1:** **RNA-binding proteins involved in plant RNA virus resistance**.

**Protein**	**Organism**	**Function**	**Target**	**RBP type**	**References**
CaPR10	*C. annuum*	Ribonuclease activity Antifungal activity Antibacterial activity	***Tobacco mosaic virus*** *Pseudomonas syringae* pv. *tomato* DC3000	RNase	Park et al., 2004; Choi et al., 2012
APUM5	*A. thaliana*	Translational repression	***Cucumber mosaic virus Turnip mosaic virus***	Pumilio homology domain (PHD)	Huh et al., 2013
AtGRP7	*A. thaliana*	RNA chaperone activity Export of mRNAs Unknown function	*Pseudomonas syringae* DC3000 *Pectobacterium carotovorum SCC1* ***Tobacco mosaic virus***	RNA-recognition motif (RRM)	Lee et al., 2012b
DRB4	*A. thaliana*	Translational repression Other unknown function	***Turnip yellow mosaic virus***	DsRNA-binding motif (dsRBP)	Jakubiec et al., 2012
BTR1	*A. thaliana*	Translational repression	***Tomato mosaic virus***	K-homology RNA-binding domain (KH)	Fujisaki and Ishikawa, 2008

### Conflict of interest statement

The authors declare that the research was conducted in the absence of any commercial or financial relationships that could be construed as a potential conflict of interest.
